# The *Arabidopsis thaliana* METACASPASE IIf Regulates Sugar Metabolism and Delays Dark‐Induced Leaf Senescence

**DOI:** 10.1111/ppl.70888

**Published:** 2026-04-21

**Authors:** Isura Sumeda Priyadarshana Nagahage, Angela Carrio‐Segui, Shashank K. Pandey, Hannele Tuominen

**Affiliations:** ^1^ Umeå Plant Science Centre, Department of Plant Physiology Umeå University Umeå Sweden; ^2^ Umeå Plant Science Centre, Department of Forest Genetics and Plant Physiology Swedish University of Agricultural Sciences Umeå Sweden

**Keywords:** *Arabidopsis thaliana*, leaf senescence, metacaspase, metacaspase‐dependent proteolysis, sugar metabolism

## Abstract

The *Arabidopsis thaliana METACASPASE IIf* (*AtMCA‐IIf*) is expressed during both developmental cell death and leaf senescence in *Arabidopsis thaliana*. It has been shown to contribute to developmental cell death but its role in regulating leaf senescence has remained unclear. To investigate this, we conducted dark‐induced senescence assays using *atmca‐IIf* mutant lines, which exhibited an accelerated senescence phenotype accompanied by increased levels of glycolytic sugars in light and slow degradation of starch in dark‐incubated leaves. Our findings therefore support *AtMCA‐IIf* function in leaf senescence through modulation of sugar metabolism. We propose, on the basis of both in vitro and in vivo data, that one of the proteolytic targets of AtMCA‐IIf in this process is PFP_β1_, a subunit of the pyrophosphate‐dependent fructose‐6‐phosphate phosphotransferase _β1_ (PFP_β1_), a key enzyme in the glycolytic pathway. Accordingly, *pfp*_*β1*_ mutants displayed accelerated senescence of dark‐incubated leaves. Together, our results suggest that AtMCA‐IIf mitigates leaf senescence through targeted modulation of sugar metabolism, thereby delaying the progression of senescence under dark conditions.

## 1 Introduction

The plant leaf accommodates both acquisition of chemical energy through photosynthesis and utilization of the energy through cellular respiration, whereby the photosynthetic products are utilized to produce energy and carbon skeletons for numerous biosynthetic pathways and nitrogen assimilation (O'Leary and Plaxton 2016). Once these functions can no longer be efficiently sustained, a process of leaf senescence is initiated to remobilize nutrients into developing seeds or storage in other parts of the plant (Hörtensteiner and Feller 2002). Leaf senescence can be part of normal plant development, but it can also be induced by environmental signals such as prolonged darkness, drought, diseases, nutrient stress, or mechanical injuries (Kim et al. 2018). Soluble sugars, such as glucose, fructose, and sucrose, function not only as energy sources but also as key signaling molecules mediating plant responses to environmental stresses (Sami et al. 2016; Ni et al. 2020; Ren et al. 2022). Leaf senescence can be triggered by both the accumulation and depletion of sugars, depending on the severity and duration of stress. Moderate abiotic stress, including short‐term drought or temperature extremes, leads to the accumulation of sugars, in particular sucrose and fructose, which negatively feeds back on photosynthesis and promotes senescence (Asad et al. 2021). In contrast, severe stress or age‐dependent decline results in sugar depletion, leading to oxidative stress and activation of sugar signaling pathways that also induce senescence (Chaomurilege et al. 2023). Two key enzymes integrating sugar metabolism and signaling, which are closely tied to leaf senescence, are hexokinase and phosphofructokinase (Dai et al. 1999; Perby et al. 2022).

Correct timing and response to the external cues require precise regulation of leaf senescence (Guo et al. 2021). While the transcriptional regulation of leaf senescence has been intensively studied during the last decades (Schippers 2015; Guo et al. 2024), the effectors and the downstream processes are not well understood. Different types of proteolytic enzymes have been implicated in bulk protein degradation during senescence (Roberts et al. 2012; Diaz‐Mendoza et al. 2014). Several members of the cysteine protease family have got large attention due to their strong transcriptional induction during senescence, and some of them, such as the *SENESCENCE ASSOSIATED GENE* (*SAG*) genes, are routinely used as markers of senescence (Lohman et al. 1994). However, it has not been possible to establish their function in senescence, possibly due to redundancy within the gene families. For instance, a mutation in the Arabidopsis *SAG12*, the most commonly used marker gene for senescence, does not show any visible senescence phenotype (Otegui et al. 2005; James et al. 2018). Even more surprisingly, mutants in the genes that give rise to the major proteolytic activities during senescence show only minor changes in whole plant senescence (Pruzinska et al. 2017).

Plant metacaspases are cysteine proteases having structural similarity with caspases that regulate the apoptotic cell death in animals (Minina et al. 2017). The *Arabidopsis thaliana* *METACASPASE IIf* (*AtMCA‐IIf*, also known as *METACASPASE 9*, AT5G04200) is well known for its specific expression in cells undergoing developmental cell death (Olvera‐Carrillo et al. 2015). *AtMCA‐IIf* is also highly expressed during the developmental death of the xylem elements, and loss of AtMCA‐IIf function results in slow xylem vessel autolysis in the primary root (Bollhöner et al. 2013). AtMCA‐IIf has also been implicated in the regulation of lateral root emergence (Stael et al. 2023) and hypocotyl elongation (Tsiatsiani et al. 2013). Recent reports have highlighted a possible role of AtMCA‐IIf also in various stress responses (Huh 2022). In pepper plants, a homolog of AtMCA‐IIf was shown to stimulate cell death in response to the virulent pathogen *Xanthomonas campestris* pv. *vesicatoria* (Kim et al. 2013). A function in senescence has been proposed on the basis of increased expression of *AtMCA‐IIf* during developmental senescence of leaves (Kwon and Hwang 2013) as well as dark‐induced senescence (Cui et al. 2013). A function in senescence is indicated also by a few other studies. ORESARA1 (ORE1), a master regulator of leaf senescence, binds to the *AtMCA‐IIf* promoter and activates it in old leaves (Balazadeh et al. 2010). Also, another transcriptional regulator of age‐dependent senescence, ANAC087, directly binds to the promoter of *AtMCA‐IIf* (Chen et al. 2023). Furthermore, *AtMCA‐IIf* expression is significantly increased in the *ANAC087* overexpression lines (Chen et al. 2023). However, functional evidence for the role of AtMCA‐IIf in leaf senescence is still missing.

The primary objective of this study was to explore the function of AtMCA‐IIf in leaf senescence. To achieve this, we induced leaf senescence in dark‐incubated, detached leaves, which facilitates fast chlorophyll degradation and protein catabolism (Weaver et al. 1998; Guo and Gan 2005). The *AtMCA‐IIf* promoter activity was induced during leaf senescence, and the *atmca‐IIf* mutant leaves displayed upregulation of senescence‐associated genes and accelerated senescence in darkness. Our results on starch and sugar metabolism in senescing leaves suggested interference of *AtMCA‐IIf* with the glycolytic pathway, which was also supported by genetic and biochemical evidence.

## 2 Materials and Methods

### 2.1 Plant Materials

The Columbia ecotype of *Arabidopsis thaliana* (Col‐0) was used as a wild‐type control for all experiments. *AtMCA‐IIf* T‐DNA insertion mutants, including *atmca‐IIf‐1* (GK‐540H06) and *atmca‐IIf‐2* (SALK_075814) reported in Bollhöner et al. (2013), as well as mutants in *FRUCTOSE‐6‐PHOSPHATE PHOSPHOTRANSFERASE β1* (AT1G12000) (*pfp*_*β1*_ *;* SALK_111562), *GLYCERALDEHYDE 3‐PHOSPHATE DEHYDROGENASE 1* (AT3G04120) (*gapc1;* SALK_129091) (Guo et al. 2012) and *ENOLASE 2* (AT2G36530) (*eno2*; SALK_021737) (Liu et al. 2020) were obtained from the Arabidopsis Biological Resource Center (ABRC). Homozygous lines were verified by PCR (Figure S1). The primers for PCR screening are listed in Table S1. *AtMCA‐IIf* overexpression lines (MC9^OE‐1^ and MC9^OE‐2^), described in Vercammen et al. (2006), were obtained from the laboratory of Frank van Breusegem. The MC9^OE‐2^ (*AtMCA‐IIf*‐OX) line was selected as a representative overexpression line due to its high overexpression level (Figure S2). Plants were grown under long‐day conditions (16/8 h light/dark; 100 μmol m^−2^ s^−1^ light intensity) at 22°C unless otherwise specified.

For genetic complementation experiments, Greengate constructs were made according to the protocols described in Lampropoulos et al. (2013). The *CAB3* (AT1G29910, −1400 to +9 bp), *IRX1* (AT4G18780, −700 to +9 bp), and *AtMCA‐IIf* (AT5G04200, −1300 to +9 bp) promoters were amplified by PCR and inserted to A000 Greengate module. The coding sequence (CDS) for the wild‐type version of *AtMCA‐IIf* was also amplified by PCR and inserted into the C000 Greengate module. Point mutagenesis of *AtMCA‐IIf* (Cys^147^/Ala) was conducted using the “megaprimer” method (Colosimo et al. 1999) and the primers listed in Table S1 to create *AtMCA‐IIf‐*C/A, an enzymatically inactive version of AtMCA‐IIf. The Greengate modules B001 (mCherry‐linker), D002 (D‐dummy), E001 (RBCS), F001 (BastaR), and Z003 (Destination vector), together with the promoter module (A000) and coding sequence module (C000), were assembled to generate the final binary vector. *atmca‐IIf‐2* mutant plants were transformed with the binary vectors containing the wild‐type *AtMCA‐IIf* and the enzymatically inactive *AtMCA‐IIf‐*C/A coding sequences using *Agrobacterium tumefaciens* (GV3101) and the floral dip method.

### 2.2 Analysis of Leaf Senescence in Detached Leaves and Leaf Discs

The experiment was conducted as previously described (Nagahage et al. 2020, 2023). For the analysis of dark‐induced leaf senescence, fifth rosette leaves (calculated from the oldest leaf) from four‐week‐old plants (unless otherwise stated) were excised and placed on moistened filter paper in petri dishes. The dishes were sealed with surgical tape, either wrapped with aluminum foil or left uncovered, and then incubated at 22°C under continuous light conditions. Chlorophyll was extracted using dimethyl sulfoxide at 60°C–65°C for 1 h. The chlorophyll extract was subsequently subjected to absorbance measurements using spectroscopy at wavelengths of 649 and 665 nm, following the procedure outlined in Wellburn (1994).

### 2.3 Histochemical GUS Staining

Leaves from four‐ to five‐week‐old, long‐day and soil‐grown pro*AtMCA‐IIf::GUS* lines, described in Bollhöner et al. (2013), were used after 5 days of dark‐induced senescence. We used the fourth to sixth rosette leaves as they naturally differ in their progression of senescence. This sampling strategy enabled us to visualize promoter activity across a spectrum of developmental stages within the same GUS line. The GUS solution consisted of 50 mM phosphate buffer at pH 7, 10 mM EDTA at pH 8, 0.24% Triton X‐100, and 2 mM X‐Gluc. Tissues were vacuum‐infiltrated with the GUS solution in three cycles of 10 min each and then incubated overnight at 37°C. The GUS solution was subsequently removed, and the leaves were rinsed with water. Next, 70% ethanol (EtOH) was added and gently agitated until the tissues bleached.

### 2.4 Starch Staining and Quantification

For starch staining, the protocol described by Fataftah et al. (2021) was followed. In brief, fifth leaves from 4‐week‐old plants grown under long‐day conditions were harvested at the end of the light cycle. Leaves were bleached with 80% ethanol (EtOH) at 80°C for 10–20 min until the chlorophyll was completely removed from the leaves. The bleached leaves were then stained with an iodine solution containing 0.7% (w/v) potassium iodide (KI) and 0.3% (w/v) iodine (I) for 3 min. The amount of starch at the end of light and dark periods was quantified as described by Smith and Zeeman (2006) and Hendriks et al. (2003). In brief, the pellets obtained from ethanol extraction were dissolved by heating to 95°C in 0.1 M NaOH for 30 min. After acidification to pH 4.9 with an HCl/sodium‐acetate mixture, a portion of the suspension underwent overnight digestion with amyloglucosidase (Roche Cat. No. 10102857001) and α‐amylase (Roche Cat. No. 10102814001). The glucose content of the resulting supernatant was then measured to determine the starch content of the sample (Roach et al. 2012).

### 2.5 Gas Exchange Measurements

The photosynthetic rate and stomatal conductance of the leaves were measured using a portable CO_2_ infrared gas analyzer (LI‐6400XT, LI‐COR Environmental) with the following conditions of the chamber: irradiance (1000 μmol photons m^−2^ s^−1^), temperature (20°C), CO_2_ concentration (400 μmol mol^−1^), and flow rate (250 cm^3^ min^−1^). Measurements were taken from the fifth leaf (without detachment) of 4‐week‐old plants grown under long‐day conditions (16/8 h light/dark; 100 μmol m^−2^ s^−1^ light intensity) at 22°C, 4 h after the start of the light cycle.

### 2.6 Metabolome Analysis

Levels of sugar phosphates were measured by mass spectrometry in the fifth leaves of 4‐week‐old plants grown in long‐day conditions (16/8 h light/dark; 100 μmol m^−2^ s^−1^ light intensity; 22°C), referred to as 0‐day samples, and in detached leaves incubated in darkness for 1, 2, and 4 days. Sugar phosphate analysis was done by referring to the Rende et al. (2019) method with modifications. Briefly, the synthetic standards for sugar phosphate analysis (2‐phosphoglyceric acid, 3‐phosphoglyceric acid, 3‐phosphoglyceraldehyde, dihydroxyacetone phosphate, erythrose‐4‐P, xylulose‐5‐P, ribulose‐5‐P, 2‐deoxyglucose (internal standard), ribose‐5‐P, glucose‐1‐P, glucose‐6‐P, mannose‐1P, mannose‐6P, fructose‐6‐P, sedoheptulose‐7‐P, trehalose‐6‐P, sucrose‐6‐P, UDP‐glucose) were all purchased from Sigma‐Aldrich, except sedoheptulose‐7‐phosphate, which was bought from Carbosynth Limited. A nine‐point calibration curve (50–20,000 pg/μL) was prepared by serial dilutions and spiked with the internal standard (2‐deoxyglucose‐6‐phosphate) at a final concentration of 250 pg/μL.

Frozen 20 mg samples were extracted in 1000 μL of ice‐cold extraction mixture consisting of chloroform:MeOH:H_2_O (1:3:1), containing 112.5 pg/μL of the internal standard 2‐deoxyglucose‐6‐phosphate, resulting in a final concentration of 250 pg/μL. The metabolites were extracted using a mixer mill set to a frequency of 30 Hz for 3 min, with 1 tungsten carbide bead added to each tube. The obtained extracts were centrifuged at 11,500 × *g* for 10 min. Two hundred microliters of the supernatant was transferred into an LC vial and evaporated to dryness using a SpeedVac.

For derivatization, dried samples were dissolved in 20 μL of methoxyamine (15 μg/μL in pyridine) and incubated on a heat block at 60°C for 30 min. After overnight incubation at room temperature, 12 μL of 1‐methylimidazole and 6 μL of propionic acid anhydride were added and heated at 37°C for 30 min. The reaction mixture was then evaporated to dryness by N2 gas. Prior to LC–MS analysis, derivatized metabolites were dissolved in 90 μL of aqueous 0.1% formic acid.

Quantitative analysis was done by combined ultra‐high‐performance liquid chromatography‐electrospray ionization‐triple quadrupole‐tandem mass spectrometry (UHPLC‐ESI‐QqQ‐MS/MS) in dynamic multiple‐reaction‐monitoring (MRM) mode. An Agilent 1290 UHPLC chromatograph equipped with a Waters Acquity BEH 1.7 μm, 2.1 × 100 mm column (Waters Corporation) coupled to an Agilent 6495 QqQ‐MS/MS (Agilent Technologies) was used. The washing solution for the autosampler syringe and injection needle was 90% MeOH with 1% HCOOH. The mobile phase consisted of A, 2% HCOOH, and B, MeOH with 2% HCOOH. The gradient was 0% B for 1 min followed by linear gradients from 0% to 30% from 1 to 3 min, then 30% to 40% B from 3 to 6 min, hold at 40% B from 6 to 10 min, followed by 40% to 70% B from 10 to 12.5 min, hold at 70% B from 12.5 to 15 min, and thereafter 70% to 99% B from 15 to 17.5 min. B was held at 99% for 0.5 min, and thereafter the column was re‐equilibrated to 0% B. The flow rate was 0.65 mL/min during equilibration and 0.5 mL/min during the chromatographic run. The column was heated to 50°C, and the injection volume was 1 μL. The mass spectrometer was operated in negative ESI mode with a gas temperature of 230°C; gas flow 12 L/min; nebulizer pressure 20 psi; sheath gas temperature 400°C; sheath gas flow 12 L/min; capillary voltage 4000 V (neg); nozzle voltage 500 V; iFunnel high pressure RF 150 V; iFunnel low pressure RF 60 V. The fragmentor voltage was 380 V, and cell acceleration voltage 4 V. Data were processed using MassHunter Qualitative Analysis and Quantitative Analysis (QqQ; Agilent Technologies) and Excel (Microsoft, Redmond) software.

### 2.7 Assessment of Protein Cleavage In Vitro

Coding sequences for the wild type (*AtMCA‐IIf*, *PFP*_*β1*_) as well as the inactive *AtMCA‐IIf*‐C/A were amplified by PCR (see Table S1). These PCR products were then cloned into pET24d in frame with a N‐terminus His‐TEV‐tag using restriction enzyme cloning. Subsequently, all plasmids were transformed into *Escherichia coli* strain Rosetta (DE3), and protein expression was induced with 0.4 mM isopropyl β‐D‐1‐thiogalactopyranoside at 20°C overnight. Cell lysis was performed by sonication, and the proteins were purified using Ni‐resin (Roche) affinity chromatography, followed by elution in a buffer containing 50 mM sodium phosphate at pH 8.0, 300 mM NaCl, and 300 mM imidazole. The His‐tag was subsequently removed by cleavage with TEV protease provided by the Protein Expertise Platform at Umeå University, Sweden. Recombinant proteins were prepared in a concentration range of 0 to 6000 nM in the optimal activity buffer for AtMCA‐IIf (50 mM MES, pH 5.5, 150 mM NaCl, 10% (w/v) sucrose, 0.1% (w/v) CHAPS, and 10 mM dithiothreitol). The recombinant proteins were incubated at 30°C for 30 min to test protein cleavage patterns. The recombinant protein for the inactive AtMCA‐IIf (rMCA‐IIf‐C/A) served as a negative control. One‐tenth of the digestion assay products was separated using SDS‐PAGE.

### 2.8 Assessment of Protein Cleavage In Vivo

For immunoblot analysis, protein quantification was performed with a DC Protein Assay Kit II #5000112 (Bio‐Rad). Crude extracts of detached, fifth leaves of long‐day grown 4‐week‐old plants, kept for 2 days in darkness, were mixed with Laemmli sampling buffer containing β‐mercaptoethanol and incubated at 95°C for 10 min before separating the protein mixtures on a reducing 12% polyacrylamide gel.

After migration at 100 V, proteins were transferred for 1 h at 270 mA onto a 0.45 μm nitrocellulose membrane. Membranes were blocked with 5% milk in Tris‐buffered saline‐Tween 20 (TBS‐T) for 1 h, followed by an overnight incubation at 4°C with a rabbit polyclonal primary antibody against the recombinant *Arabidopsis thaliana* PFP_β1_ (PFP_β1_; 1/500, Biorbyt), diluted in 2% milk in TBS‐T.

After a 1‐h incubation at room temperature with goat anti‐rabbit secondary antibody conjugated to horseradish peroxidase (1/10,000 in 2% milk in TBS‐T, Agrisera, AS09 602), visualization was carried out using a chemiluminescence kit (Agrisera ECL kit bright/Super bright; AS16 ECL‐SN). For normalization, Histone H3 was detected using a rabbit polyclonal anti‐H3 antibody (H3; 1/10,000, Agrisera) as a loading control. AtMCA‐IIf was detected using the antibody described in Vercammen et al. (2006). Signals were detected using the Azure c600 Western Blot Imaging system (Azure Biosystems).

### 2.9 qRT‐PCR Analysis

Total RNA was extracted from detached, fifth rosette leaves of 4‐week‐old, long‐day‐grown plants incubated for 0, 3, 5, and 6 days in darkness using Spectrum Plant total RNA kit (Sigma) together with On‐Column DNase I Digest Set (Sigma). Reverse transcription and qRT‐PCR analyses were performed as described in (Carrió‐Seguí et al. 2016). The *UBIQUITIN10*, *ACTIN2*, and *GAPDH* genes were used as reference genes for data normalization. Four biological replicates were used, each analyzed in two technical replicates, and the mean ratios ± SE were calculated.

## 3 Results

### 3.1 *AtMCA‐IIf* Promoter Is Active During Dark‐Induced Senescence

**FIGURE 1:**
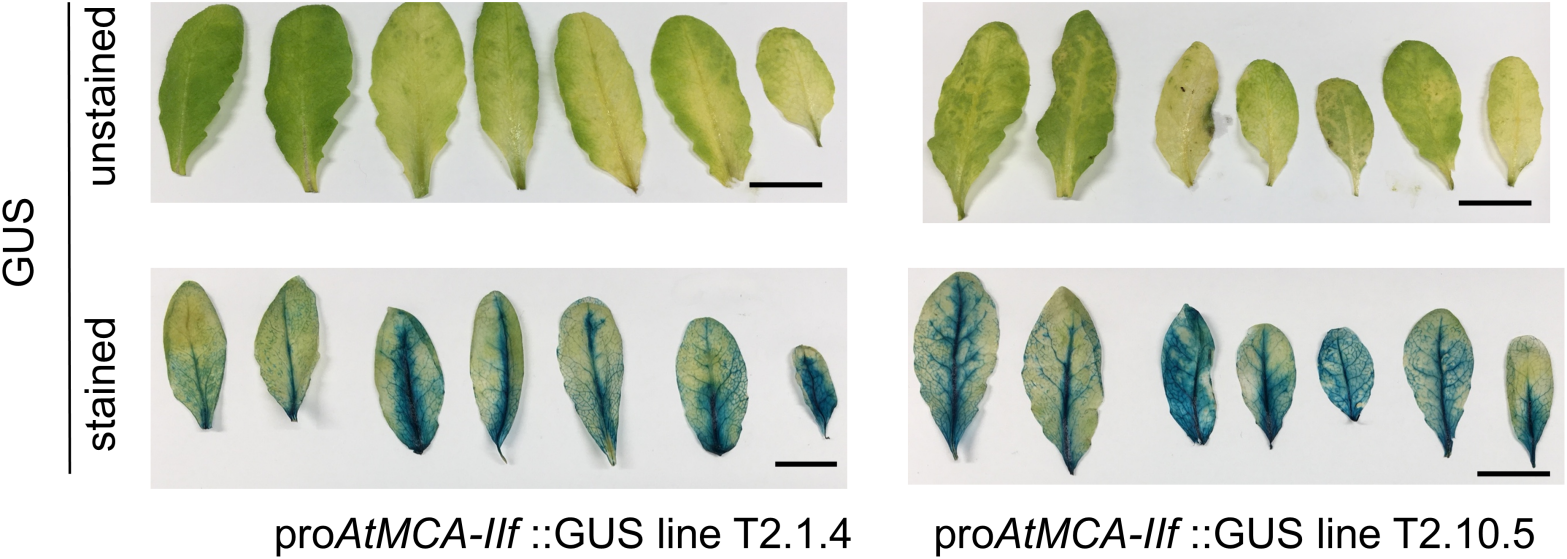
Promoter activity of *AtMCA‐IIf* in dark‐incubated leaves. The fourth to sixth rosette leaves were detached from long‐day‐grown, 4‐week‐old plants and kept in darkness for 5 days according to Nagahage et al. (2020). The upper row shows unstained leaves, and the lower row shows leaves stained for the activity of the 1410‐bp *AtMCA‐IIf* promoter fused to β‐glucuronidase (GUS) reporter gene in two transgenic lines. Bar = 1 cm.

To investigate AtMCA‐IIf involvement in leaf senescence, we first analyzed *AtMCA‐IIf* promoter activity in *GUS* lines under different light conditions. Leaves incubated under continuous light conditions did not exhibit clear GUS staining (Figure S3). Next, we conducted a dark‐induced senescence experiment and characterized leaves for both the progression of senescence and the GUS activity. After 5 days of dark conditions, when the leaves were already yellowish and hence at the late stage of senescence, *AtMCA‐IIf* promoter activity was visible along the veins and also in between the veins (Figure 1). Interestingly, we observed a good spatial overlap between the areas in the leaves with the most advanced senescence and the highest GUS signal (Figure 1), suggesting involvement of AtMCA‐IIf in the regulation of dark‐induced senescence.

### 3.2 AtMCA‐IIf Negatively Regulates Dark‐Induced Leaf Senescence

**FIGURE 2:**
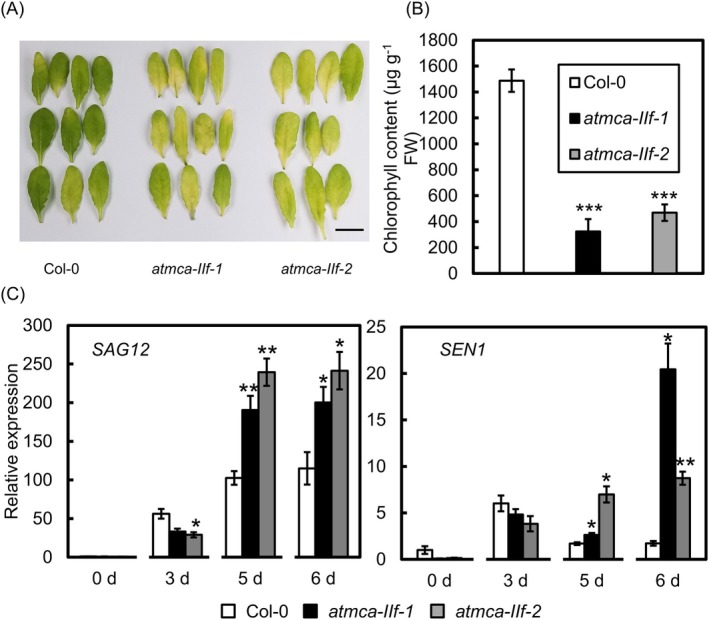
The function of AtMCA‐IIf in dark‐induced senescence of detached leaves. (A) The progress of senescence in Col‐0 wild type and *atmca‐IIf* mutant leaves incubated in darkness for 5 days. Bar = 1 cm. (B) Chlorophyll content in the detached leaves incubated in darkness for 5 days. Values and error bars indicate means ± SE (*n* = 5 biologically independent replicates). Asterisks indicate means that are significantly different from Col‐0 wild type (Welch's *t*‐test (two‐tailed) ***p < 0.001). (C) The expression of leaf senescence‐associated genes *SENESCENCE‐ASSOCIATED GENE 12* (*SAG12*) and *SENESCENCE 1* (*SEN1*) in Col‐0 wild type and *atmca‐IIf* leaves. Quantitative RT‐PCR analysis was performed using detached leaves incubated for 0, 3, 5, and 6 days in darkness. Values and error bars indicate means ± SE (*n* = 4 biologically independent replicates). Asterisks indicate means that are significantly different from the Col‐0 wild type (Welch's *t*‐test (two‐tailed) **p < 0.01, *p < 0.05). In each case, the 5th rosette leaves (calculated from the oldest leaf) were excised from 5‐week‐old plants and placed on moistened filter paper in petri dishes that were sealed and covered with aluminum foil for the duration of the experiment.

To verify the role of AtMCA‐IIf in leaf senescence, we conducted dark‐induced senescence experiments with detached leaves of *atmca‐IIf* mutants and a transgenic line overexpressing *AtMCA‐IIf* under the control of the *CaMV35S* promoter (*AtMCA‐IIf‐*OX). No differences were present in continuous light conditions (Figure S4A). Also, the photosynthetic rate and stomatal conductance were similar between the *atmca‐IIf* mutants and the Col‐0 wild‐type plants in light (Figure S5). However, under continuous dark conditions, leaf senescence progressed more rapidly in the leaves of the *atmca‐IIf* mutants compared to the Col‐0 wild type, while the leaves of the *AtMCA‐IIf‐*OX line remained similar to the wild type (Figures 2A and S4A). The chlorophyll content of the different genotypes followed the same pattern (Figures 2B and S4B). Also, the expression of senescence marker genes supported enhanced senescence in the *atmca‐IIf* mutant plants. The expression levels of both *SENESCENCE‐ASSOCIATED GENE 12* (*SAG12*) and *SENESCENCE 1* (*SEN1*) were significantly higher in the *atmca‐IIf* mutant plants compared to Col‐0 in leaves incubated five and 6 days in darkness (Figure 2C). These results demonstrate that AtMCA‐IIf suppresses dark‐induced leaf senescence.

To assess whether this effect also applies to age‐dependent senescence, we performed an additional experiment using the *atmca‐IIf‐2* mutant line under normal long‐day conditions (Figure S6). With a regular watering schedule (every 2 days), no apparent differences were seen between the mutant and Col‐0 plants. However, under reduced watering frequency (every 5 days), which introduced mild drought stress, the *atmca‐IIf‐2* mutant plants showed accelerated senescence compared to wild‐type plants (Figure S6). These findings suggest that AtMCA‐IIf may have a broader regulatory role in senescence that is conserved across multiple pathways.

Complementation experiments were conducted in the *atmca‐IIf‐2* mutant background (Figure S7), followed by senescence experiments in detached fifth leaves of 4‐week‐old plants incubated for 4 days under dark conditions. Expression of the enzymatically active *AtMCA‐IIf* under the control of the endogenous promoter reverted the *atmca‐IIf*‐2 mutant senescence phenotype in two out of three independent lines, while the expression of the enzymatically inactive *AtMCA‐IIf* C/A did not (Figure S7A–C). It also seemed that the expression of the enzymatically active *AtMCA‐IIf* specifically in the xylem vessels, under the control of the *IRX1* promoter, as well as in the mesophyll cells, under the control of the *CAB3* promoter, could complement the *atmca‐IIf*‐*2* senescence phenotype. There was not always a perfect correlation between the expression level of *AtMCA‐IIf* and the extent of complementation (Figure S7F), which could result from position effects of transgene expression or failure to capture the timing of expression required for functional complementation during senescence. Additionally, previous studies have shown that overexpression of proteases, including plant metacaspases, can result in dominant or pleiotropic phenotypes, which complicate functional interpretation, rather than simply reflecting their physiological roles (van der Hoorn 2008; Pitsili et al. 2023). As an example of the latter possibility, the highest expression of AtMCA‐IIf in the xylem vessels in line N49‐15 resulted in dwarf plants without complementation. Importantly, all other lines having a good expression level of *AtMCA‐IIf* complemented the phenotype (Figure S7D–F). Taken together, these results support the function of the enzymatically active AtMCA‐IIf in leaf senescence, and that the expression in either the vessel elements or the mesophyll cells is sufficient for this function.

### 3.3 AtMCA‐IIf Influences the Accumulation of Sugar Phosphates

**FIGURE 3:**
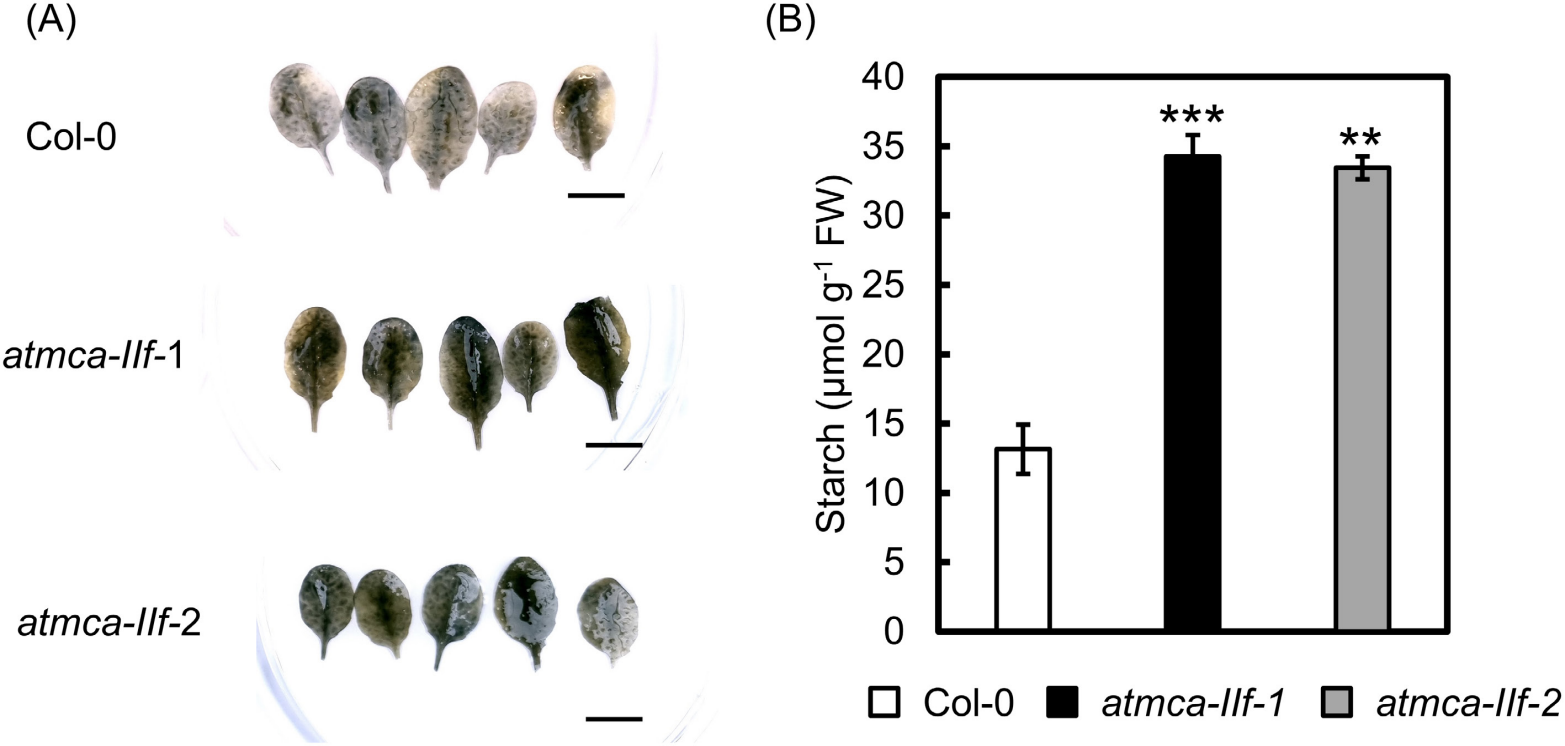
The effect of AtMCA‐IIf on starch content of dark‐incubated, detached leaves. (A) Iodine staining of starch in detached, dark‐incubated leaves. Bar = 1 cm. (B) The starch content in detached, dark‐incubated leaves. Values and error bars indicate means ± SE (*n* = 3 biologically independent replicates). In each case, the fifth rosette leaves were excised from four‐week‐old plants and placed on moistened filter paper in petri dishes that were sealed and covered with aluminum foil for 2 days. Asterisks indicate means that are significantly different from Col‐0 wild type (Welch's *t*‐test (two‐tailed) ***p < 0.001, **p < 0.01).

Accelerated senescence has been linked to impaired degradation of starch stored in leaves and subsequently degraded during the night to support plant growth and development (Garapati et al. 2015). To test whether the *atmca‐IIf* mutants exhibit any alterations in starch metabolism, we analyzed the starch content in detached leaves of *atmca‐IIf* mutant and the *AtMCA‐IIf* overexpression line in light and darkness. At the end of the light cycle of plants grown in long‐day conditions (referred to as 0 days), the leaves of the *atmca‐IIf* mutant did not show altered levels of starch, but those of the *AtMCA‐IIf* overexpression line had a lower amount of starch (Figure S8). Subsequent dark incubation of detached leaves induced senescence and led to starch degradation (Figure S8B). Interestingly, the degradation of starch was much less efficient (Figure 3) and slower (Figure S8B) in the dark‐incubated *atmca‐IIf* mutant leaves compared to the Col‐0 wild type.

**FIGURE 4:**
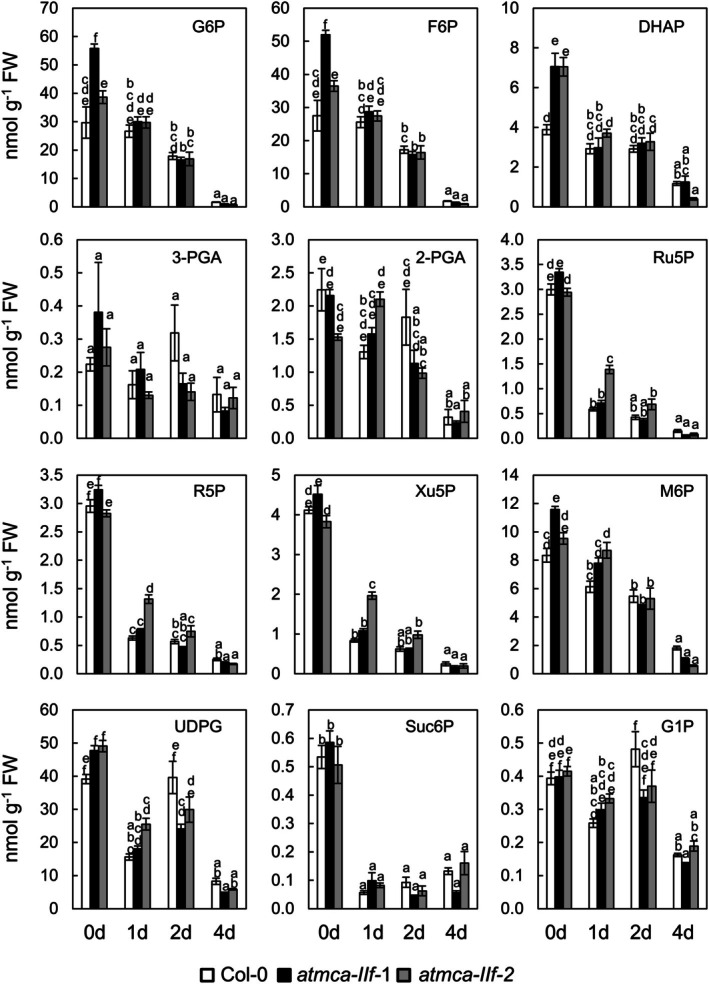
The effect of AtMCA‐IIf on the content of sugar phosphates in detached, dark‐incubated leaves. The fifth rosette leaves were excised from long‐day grown, 4‐week‐old plants and either analyzed directly, referred to as 0‐day samples, or after incubation in darkness by placing them on moistened filter paper in petri dishes that were sealed and covered with aluminum foil for 1, 2, and 4 days. Values and error bars indicate means ± SE (*n* = 4 biologically independent replicates), and different letters indicate a significant difference determined by one‐way ANOVA with Tukey's honestly significant difference test (*p* < 0.05). G6P, glucose 6‐phosphate; F6P, fructose 6‐phosphate; DHAP, dihydroxyacetone phosphate; 3‐PGA, 3‐phosphoglycerate; 2‐PGA, 2‐phosphoglycerate; Ru5P, ribulose 5‐phosphate; R5P, ribose 5‐phosphate; Xu5P, xylulose 5‐phosphate; M6P, mannose 6‐phosphate; UDPG, uridine diphosphate glucose; Suc6P, sucrose‐6‐phosphate; G1P, glucose 1‐phosphate.

As starch degradation is strictly linked to plants' sugar metabolism, impaired starch degradation is likely to coincide with changes in sugar homeostasis. We therefore analyzed the levels of sugar phosphates in leaves harvested at the end of the light cycle in long day growth conditions, referred to as 0‐day samples, and in detached leaves after incubation for 1, 2, and 4 days in darkness (Table S2). It should be noted that after 4 days of darkness, the detached leaves of the *atmca‐IIf* mutants were already senescent while the Col‐0 leaves were still green. Interestingly, glucose 6‐phosphate (G6P), fructose 6‐phosphate (F6P), dihydroxyacetone phosphate (DHAP), mannose 6‐phosphate (M6P), and uridine diphosphate glucose (UDPG) contents were higher in the 0‐day samples of the *atmca‐IIf* mutants compared to Col‐0 while the levels of these sugar phosphates reduced to more or less the same level with Col‐0 in response to 1 and 2 days of dark incubation (Figure 4). Most of these sugar phosphates are either intermediates or immediate products of the glycolytic pathway. The levels of 3‐phosphoglycerate (3‐PGA), phosphoglycerate (2‐PGA), sucrose 6‐phosphate (Suc6P), ribose 5‐phosphate (R5P), xylulose 5‐phospate (Xu5P) or glucose 1‐phosphate (G1P) did not show any consistent differences between the different genotypes (Figure 4).

The slower degradation of starch and the alterations in the abundance of the glycolytic sugar phosphates G6P, F6P, DHAP, and M6P suggest that AtMCA‐IIf interferes with the glycolytic pathway. We hypothesize that this function is critical during the night as starch degradation normally takes place during the night and that the *AtMCA‐IIf* expression in leaves occurs only in darkness (Figure 1). This hypothesis was supported by reversion of the accelerated senescence of *atmca‐IIf* mutants by incubation of leaf discs in darkness with F6P and, to some extent, with its downstream product fructose 1,6‐bisphosphate (FBP), but not by sucrose or the glycolytically inactive 2‐deoxy‐D‐glucose (Figure S9C).

### 3.4 A Mutant in a Phosphofructokinase Shows Accelerated Senescence in Darkness

**FIGURE 5:**
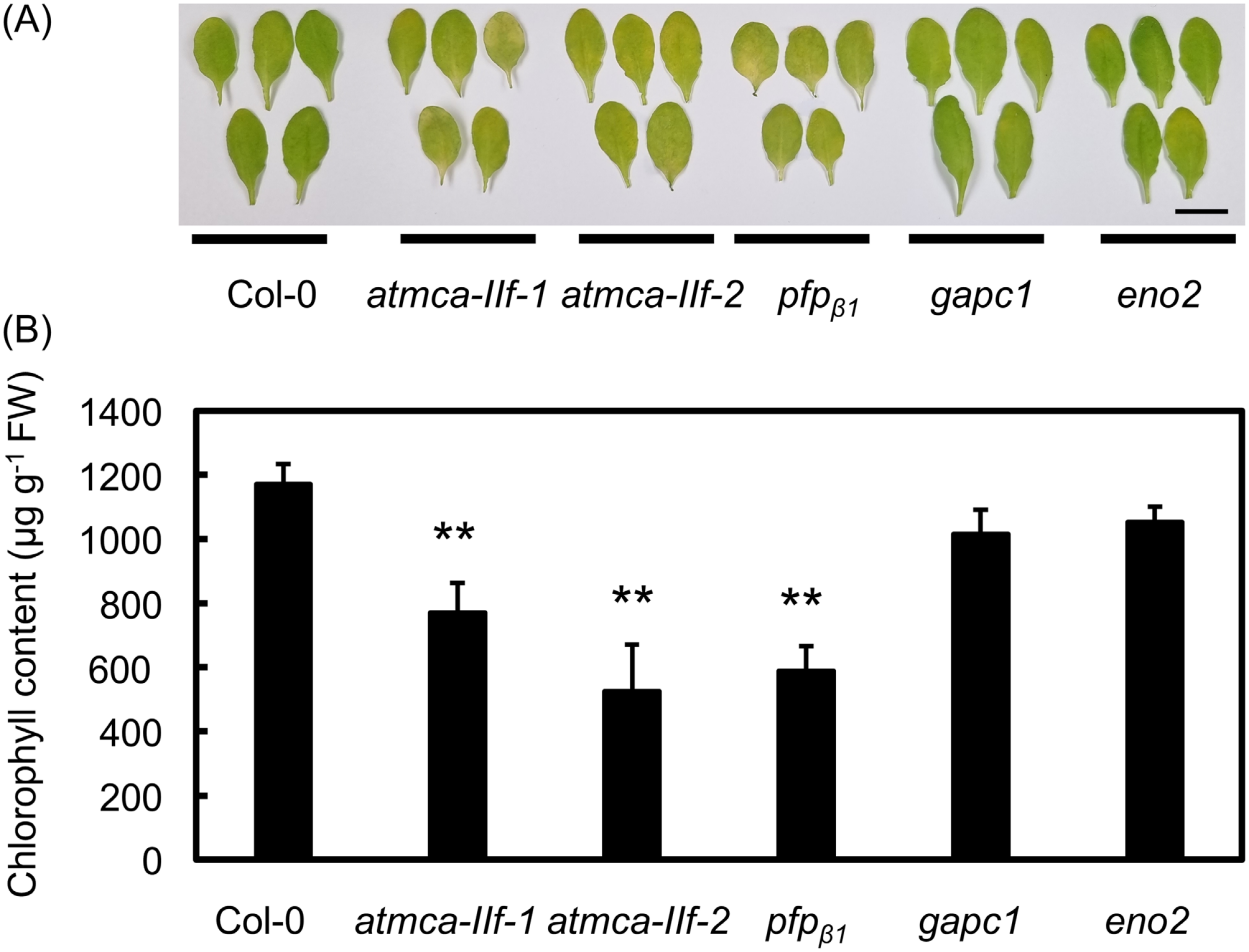
Leaf senescence in mutants of the glycolytic pathway. (A) Detached, dark‐incubated leaves of *atmca‐IIf‐1*, *atmca‐IIf‐2*, *fructose‐6‐phosphate phosphotransferase* (*pfp*_*β1*_), *glyceraldehyde 3‐phosphate dehydrogenase 1* (*gapc1*), and *enolase 2* (*eno2*) mutants. Bar = 1 cm. (B) Chlorophyll content in the detached, dark‐incubated leaves of the mutants. In each case, the fifth leaves of 4‐week‐old, long‐day grown mutant plants were detached and placed on moistened filter paper in petri dishes that were sealed and covered with aluminum foil for 4 days. Values and error bars indicate means ± SE (*n* = 5 biologically independent replicates). Asterisks indicate means that are significantly different from the Col‐0 wild type (Welch's *t*‐test (two‐tailed) **p < 0.01).

Within the glycolytic pathway, the conversion of fructose 6‐phosphate (F6P) to fructose 1,6‐bisphosphate (FBP) is considered the key rate‐limiting reaction catalyzed by phosphofructokinase (Plaxton 1996). A degradomic analysis in Arabidopsis (Tsiatsiani et al. 2013) as well as a proteomic analysis in *Populus* stem tissues (Bollhöner et al. 2018) indicated that a phosphofructokinase family protein PFP_β1_, a subunit of the pyrophosphate‐dependent fructose‐6‐phosphate phosphotransferase (PFP) enzyme, is a proteolytic target of AtMCA‐IIf. We therefore tested whether a mutant in the *PFP*_*β1*_ locus, together with two other mutants in the glycolytic pathway, shows an accelerated senescence phenotype similar to *atmca‐IIf* mutants (Figures 5 and S1). Consistent with our previous data, detached *atmca‐IIf* mutant leaves showed an accelerated senescence phenotype in darkness compared to the Col‐0 (Figure 5). Interestingly, *pfp*_*β1*_ mutant leaves also exhibited an accelerated senescence phenotype, whereas mutants in *GLYCERALDEHYDE 3‐PHOSPHATE DEHYDROGENASE 1* (*GAPC1*) or *ENOLASE 2* (*ENO2*) didn't show any clear differences compared to the Col‐0 (Figure 5). These results suggest that malfunction of PFP might lead to the accelerated senescence phenotype of the *atmca‐IIf* mutants.

### 3.5 AtMCA‐IIf Targets PFP_β1_ Both In Vitro and In Vivo

**FIGURE 6:**
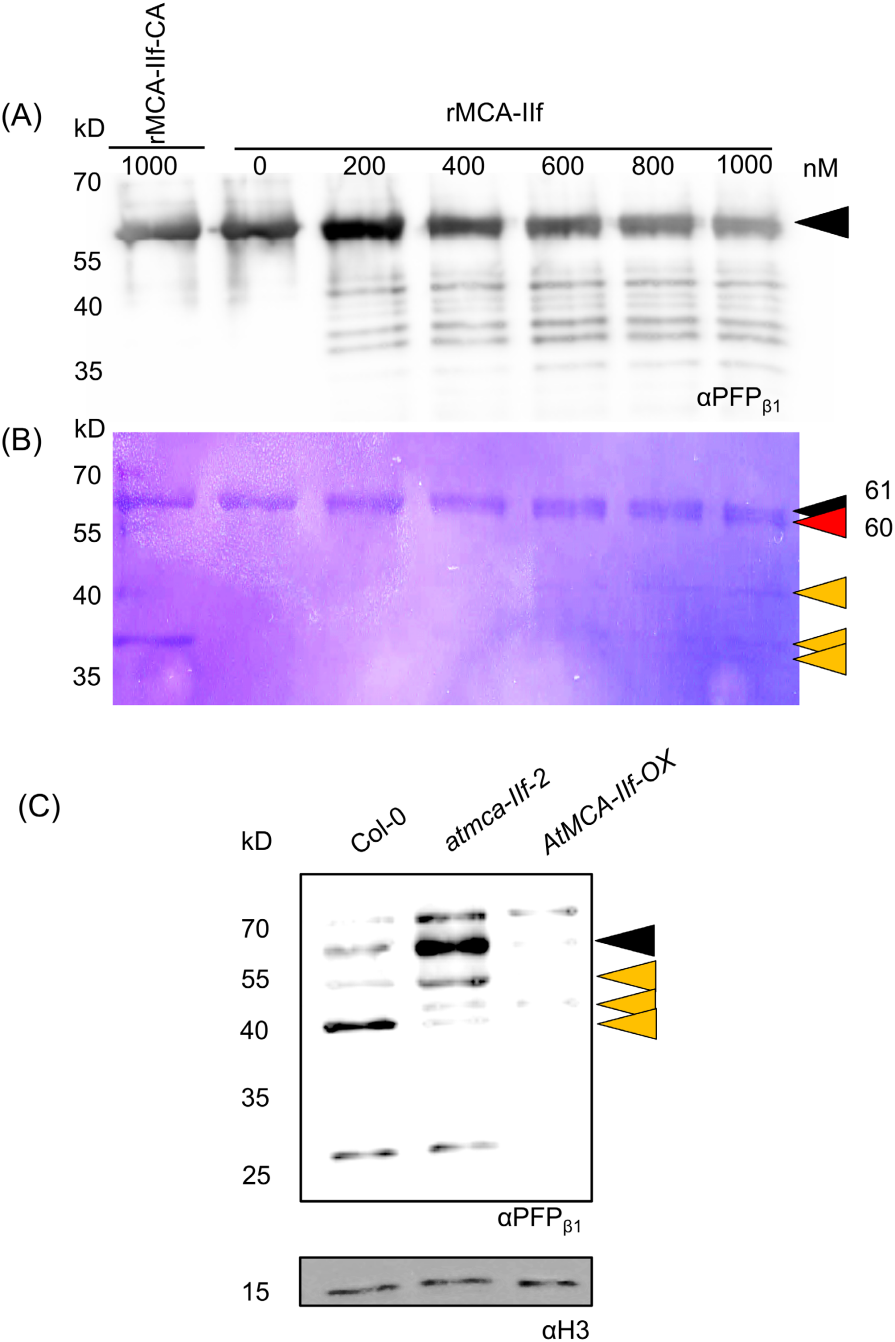
Proteolytic cleavage of PFP_β1_ by AtMCA‐IIf. (A) In vitro cleavage Assay. Recombinant PFP_β1_ was incubated with increasing amounts of recombinant AtMCA‐IIf or its inactive mutant (rMCA‐IIf‐CA), where the active‐site cysteine was mutated to alanine. The proteins were detected by western blotting using antibody against PFP_β1_. The size of the intact recombinant PFP_β1_ (61 kDa) is indicated by a black arrowhead. (B) Coomassie‐stained image for the gel used for the western blot in (A) as a loading control. The size of the intact recombinant PFP_β1_ (61 kDa) is indicated by a black arrowhead, and the putative N‐terminally cleaved PFP_β1_ (60 kDa) by a red arrowhead. Additional proteolytic fragments are indicated by orange arrowheads. (C) PFP_β1_ fragmentation pattern in vivo. Proteins were loaded from dark‐incubated fifth leaves of four‐week‐old Col‐0, *atmca‐IIf‐2* mutant and *AtMCA‐IIf‐*overexpressor (*AtMCA‐IIf*‐OX) plants. The PFP_β1_ fragmentation pattern was detected in a western blot using an antibody against PFP_β1_. A histone 3 antibody (αH3) was used as a loading control. The size of the full‐length PFP_β1_ (61 kDa) is indicated by a black arrowhead. Additional proteolytic fragments are indicated by orange arrowheads. The ~70 kDa band likely represents a nonspecific signal and may correspond to a homologous protein.

To validate the hydrolytic action of AtMCA‐IIf on PFP_β1_, we synthesized recombinant proteins for the active form of AtMCA‐IIf, the inactive form AtMCA‐IIf‐C/A, and PFP_β1_. Increasing amounts of recombinant AtMCA‐IIf reduced the level of the full‐length PFP_β1_ in vitro and increased the amount of smaller molecular weight fragments of PFP_β1_, while no cleavage was observed by the inactive AtMCA‐IIf‐C/A (Figure 6A). The Coomassie‐stained blot revealed two fragments corresponding to the expected size of the full‐length PFP_β1_ (61 kDa) and a slightly smaller fragment (~60 kDa) after incubation with the highest concentration of the recombinant AtMCA‐IIf (Figure 6B), which could correspond to the cleavage at the PFP_β1_ residue R10, identified in Tsiatsiani et al. (2013), resulting in two fragments of the sizes of 1 kDa and 60 kDa.

In vivo, PFP_β1_ was more abundant in the dark‐incubated leaves of *atmca‐IIf‐2* mutant and less abundant in the *AtMCA‐IIf‐*OX line compared to Col‐0 (Figure 6C). Also, the cleavage pattern differed between the genotypes, suggesting either additional cleavage of PFP_β1_ by AtMCA‐IIf or subsequent cleavage by other proteases. In any case, the in vivo data demonstrated that the correct processing of PFP_β1_ depends on the activity of AtMCA‐IIf.

## 4 Discussion

### 4.1 AtMCA‐IIf Regulates Dark‐Induced Senescence

AtMCA‐IIf is known to be involved in xylem development, gluconeogenesis, and autophagy. We report here a novel function for AtMCA‐IIf in leaf senescence based on accelerated senescence of the *atmca‐IIf* mutant leaves in darkness (Figure 2). Two upstream regulators of *AtMCA‐IIf* expression, ORE1 and ANAC087, were earlier shown to control senescence (Balazadeh et al. 2010; Chen et al. 2023), and our results support the function of AtMCA‐IIf as one of the downstream targets of these transcription factors in leaf senescence.

An interesting question is whether AtMCA‐IIf regulates senescence in a non‐cell autonomous manner from within the xylem vessel elements where it is expressed during developmental cell death (Bollhöner et al. 2013) or whether AtMCA‐IIf function in senescence is mediated in a cell‐autonomous manner in the senescing mesophyll cells. The latter was supported by the expansion of *AtMCA‐IIf* promoter activity from the vascular cells to the whole leaf blade during senescence (Figure 1). However, complementation of the enhanced senescence phenotype of the *atmca‐IIf* mutant was not possible only by the expression of *AtMCA‐IIf* in the leaf mesophyll cells but also when expressed specifically in the xylem vessels (Figure S7). This means that dark‐induced senescence throughout the whole leaf can be influenced by *AtMCA‐IIf* expression in the xylem vessel elements. The underlying mechanism remains unclear, but could be related to prevention or delay of spreading cell death during leaf senescence from within the xylem vessel elements, similar to what was observed earlier in Arabidopsis tracheary element cell cultures, where *AtMCA‐IIf* expression in the tracheary elements was required to prevent death of the surrounding parenchymatic cells by modulating autophagy (Escamez et al. 2016).

### 4.2 AtMCA‐IIf Targets PFP_β1_

Our data suggest that AtMCA‐IIf functions as a regulatory protease during senescence by cleaving and modulating the activity of metabolic enzymes in the glycolytic pathway. The accumulation of the sugar phosphates G6P, F6P, DHAP, and M6P in the *atmca‐IIf* mutant leaves pointed to possible defects in the enzymatic activity of PFP, leading to accumulation of the upstream glycolytic products (Figure 4). Similar sugar profiles were found in sugarcane downregulated for PFP_β_ (van der Merwe et al. 2010). These details suggested that PFP is a proteolytic target of AtMCA‐IIf. Indeed, our in vitro and in vivo experiments supported the function of AtMCA‐IIf in proteolytic processing of PFP_β1_ (Figure 6). PFP is an important enzyme in glycolysis that influences equilibration of hexose‐phosphate and triose‐phosphate pools (Hajirezaei et al. 1993), having an impact on plant energy metabolism as well as adaptation to various stress conditions (Van Schaftingen and Hers 1983; Theodorou et al. 1992; Mutuku and Nose 2012; Mustroph et al. 2013; Lim et al. 2014). Our results demonstrate a role for PFP_β1_ also in dark‐induced senescence (Figure 5). Notably, the *atmca‐IIf* and *pfp*_*β1*_ mutants exhibited a similar accelerated senescence phenotype, implying that the cleavage of PFP_β1_ by AtMCA‐IIf is required for its normal function. This parallels previous findings where metacaspases activate the function of their proteolytic targets rather than simply degrade them (Sundström et al. 2009; Tsiatsiani et al. 2013). Interestingly, another glycolytic pathway enzyme, glyceraldehyde 3‐phosphate dehydrogenase (GAPC), is a target of metacaspases (Silva et al. 2011; Escamez et al. 2019). In young Arabidopsis seedlings, AtMCA‐IIf was shown to proteolytically process also PEPCK1, a key enzyme in gluconeogenesis leading to sugar biosynthesis (Tsiatsiani et al. 2013). Notably, GAPC, PEPCK1, and PFP_β1_ are all cytosolic enzymes, and AtMCA‐IIf has also been reported to localize to the cytoplasm (Bollhöner et al. 2013; Tsiatsiani et al. 2013), supporting the plausibility of direct proteolytic regulation. Therefore, AtMCA‐IIf seems to be involved in regulating multiple steps of plant energy metabolism.

### 4.3 Glycolytic Pathway in the Crossroads of Different Energetic Choices During Senescence

**FIGURE 7:**
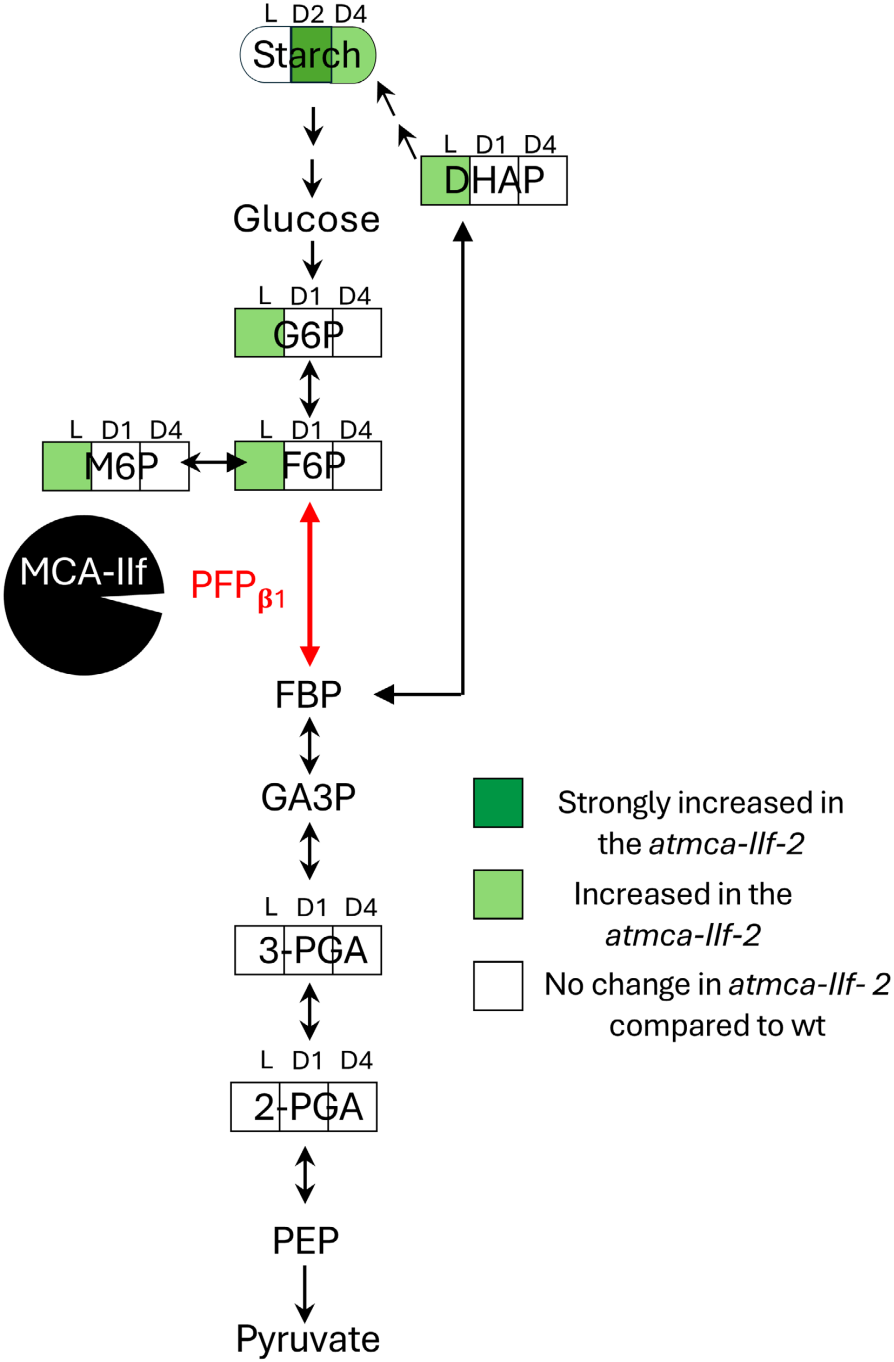
AtMCA‐IIf function during leaf senescence. AtMCA‐IIf processes PFP_β1_. When AtMCA‐IIf is absent, upstream glycolytic substrates accumulate and inhibit starch degradation in darkness. Metabolite levels at day 0 under light conditions (L), and at day 1 (D1) and day 4 (D4) in darkness during senescence induction are indicated by the color scale. The color code indicates roughly the changes in the metabolite levels in *atmca‐IIf* compared to Col‐0 wild‐type plants, with increased intensity of the colors indicating increases in the differences of the levels between *atmca‐IIf* and Col‐0. G6P, glucose 6‐phosphate; F6P, fructose 6‐phosphate; DHAP, dihydroxyacetone phosphate; 3‐PGA, 3‐phosphoglycerate; 2‐PGA, 2‐phosphoglycerate; M6P, mannose 6‐phosphate; PEP, phosphoenolpyruvate.

In addition to the accumulation of sugar phosphates, the *atmca‐IIf* mutants displayed slow degradation of starch in darkness (Figures 3 and S8). It is well established that high G6P and F6P content of leaves inhibits starch degradation during the night (Gerhardt et al. 1987; Sharkey and Weise 2016), and it is therefore possible that the slow starch degradation in dark‐incubated *atmca‐IIf* mutant leaves is due to the high amount of the sugar metabolites that accumulate during the day. Furthermore, since sugar phosphates can be utilized for respiration and other cellular processes during the night, it is possible that these sugar metabolites, rather than starch, provide the energy and metabolites needed in darkness in the *atmca‐IIf* mutant. Indeed, these metabolites dramatically decreased during prolonged dark incubation of the *atmca‐IIf* mutant leaves (Figure 4). Even though *atmca‐IIf* mutants do not show any visible phenotype in normal growth conditions, it is likely that the poor supply of sugars from starch leads, during prolonged darkness, to sugar starvation and leaf senescence. Based on these results, we propose that AtMCA‐IIf serves to safeguard plants' energy demand in two different ways (Figure 7). In normal light conditions, AtMCA‐IIf function is required for energy production through glycolysis/TCA cycle, likely through its hydrolytic action on PFB_β1_ but also via several other steps in the pathway. In stressful conditions, such as prolonged darkness, AtMCA‐IIf seems to be required for maintaining the sugar supply through starch degradation.

## Author Contributions

I.S.P.N. conceived and designed the experiments, I.S.P.N. performed most of the experiments. I.S.P.N., A.C.‐S., and S.K.P. performed the qRT‐PCR analysis. I.S.P.N., A.C.‐S., S.K.P., and H.T. analyzed and interpreted the data. I.S.P.N. and H.T. contributed reagents, materials and analytical tools. I.S.P.N. and H.T. wrote and edited the manuscript.

## Funding

This work was supported by the Vetenskapsrådet (2020‐03799) and the Knut och Alice Wallenbergs Stiftelse (2016.0352, 2018.0026, 2020.0240).

## Conflicts of Interest

The authors declare no conflicts of interest.

## Supporting information


**Figure S1:** Genotyping of the *pfp*_*𝛽1*_, *gapc1, eno2* T‐DNA insertion lines.
**Figure S2:** Immunoblot analysis of AtMCA‐IIf in crude protein extracts from fifth leaves of four‐week‐old plants incubated in darkness for 2 days.
**Figure S3:** The activity of the *AtMCA‐IIf* promoter in leaves treated in either light or darkness.
**Figure S4:** The function of AtMCA‐IIf in leaves incubated in darkness and in continuous light.
**Figure S5:** Photosynthetic rate and stomatal conductance of plants under light conditions.
**Figure S6:** Effect of *AtMCA‐IIf* on age‐dependent leaf senescence.
**Figure S7:** Leaf senescence in genetic complementation lines of the *atmca‐IIf‐2* mutant.
**Figure S8:** The effect of AtMCA‐IIf on starch content of leaves before and after the dark treatment.
**Figure S9:** The effect of fructose phosphates on the accelerated senescence phenotype of *atmca‐IIf* in darkness.

**Table S1:** Oligonucleotides used in this study.

**Table S2:** Raw data for the quantities of sugar phosphates in dark‐incubated leaves of Col‐0, and the *atmca‐IIf* mutant plants. The sugar phosphates that were analyzed were 2‐phosphoglyceric acid (2‐PGA), 3‐phosphoglyceric acid (3‐PGA), dihydroxyacetone phosphate (DHAP), Erythrose‐4P, Xylulose‐5P, Ribulose‐5P, Ribose‐5P, Glucose‐1P, Mannose‐1P, Fructose‐6P, Glucose‐6P, Mannose‐6P, Sedoheptulose‐7P, Trehalose‐6P, Sucrose‐6P and UDP‐glucose.

## Data Availability

Data supporting the findings of this study are available from the corresponding author upon request.
