# Supplementary material for: The Arabidopsis thaliana METACASPASE IIf Regulates Sugar Metabolism and Delays Dark‐Induced Leaf Senescence
Source: Physiol Plant. 2026 Apr 21;178(2):e70888. doi: 10.1111/ppl.70888 (PMC13096987; doi:10.1111/ppl.70888)
Supplement: Supplementary file 1 — Figure S1: Genotyping of the pfp 𝛽1 , gapc1, eno2 T‐DNA insertion lines. Figure S2: Immunoblot analysis of AtMCA‐IIf in crude protein extracts from fifth leaves of four‐week‐old plants incubated in darkness for 2 days. Figure S3: The activity of the AtMCA‐IIf promoter in leaves treated in either light or darkness. Figure S4: The function of AtMCA‐IIf in leaves incubated in darkness and in continuous light. Figure S5: Photosynthetic rate and stomatal conductance of plants under light conditions. Figure S6: Effect of AtMCA‐IIf on age‐dependent leaf senescence. Figure S7: Leaf senescence in genetic complementation lines of the atmca‐IIf‐2 mutant. Figure S8: The effect of AtMCA‐IIf on starch content of leaves before and after the dark treatment. Figure S9: The effect of fructose phosphates on the accelerated senescence phenotype of atmca‐IIf in darkness. [file PPL-178-e70888-s003.pdf]

SUPPLEMENTALS

The *Arabidopsis thaliana* METACASPASE IIf regulates sugar metabolism and delays dark-induced leaf senescence

Isura Sumeda Priyadarshana Nagahage[a,c*], Angela Carrio-Segui[b,d], Shashank K. Pandey[b], Hannele Tuominen[b]

[a] Umeå Plant Science Centre, Department of Plant Physiology, Umeå University, 901 87 Umeå, Sweden

[b] Umeå Plant Science Centre, Department of Forest Genetics and Plant Physiology, Swedish University of Agricultural Sciences, 901 83 Umeå, Sweden

[c] Present Address: Department of Plant and Soil Sciences, University of Kentucky, Lexington, KY 40546, USA.

[d] Present Address: Institute of Biotechnology, OEB department, University of Helsinki, 00790 Helsinki, Finland

* Corresponding author

Isura Sumeda Priyadarshana Nagahage, E-mail: isura.nagahage@uky.edu

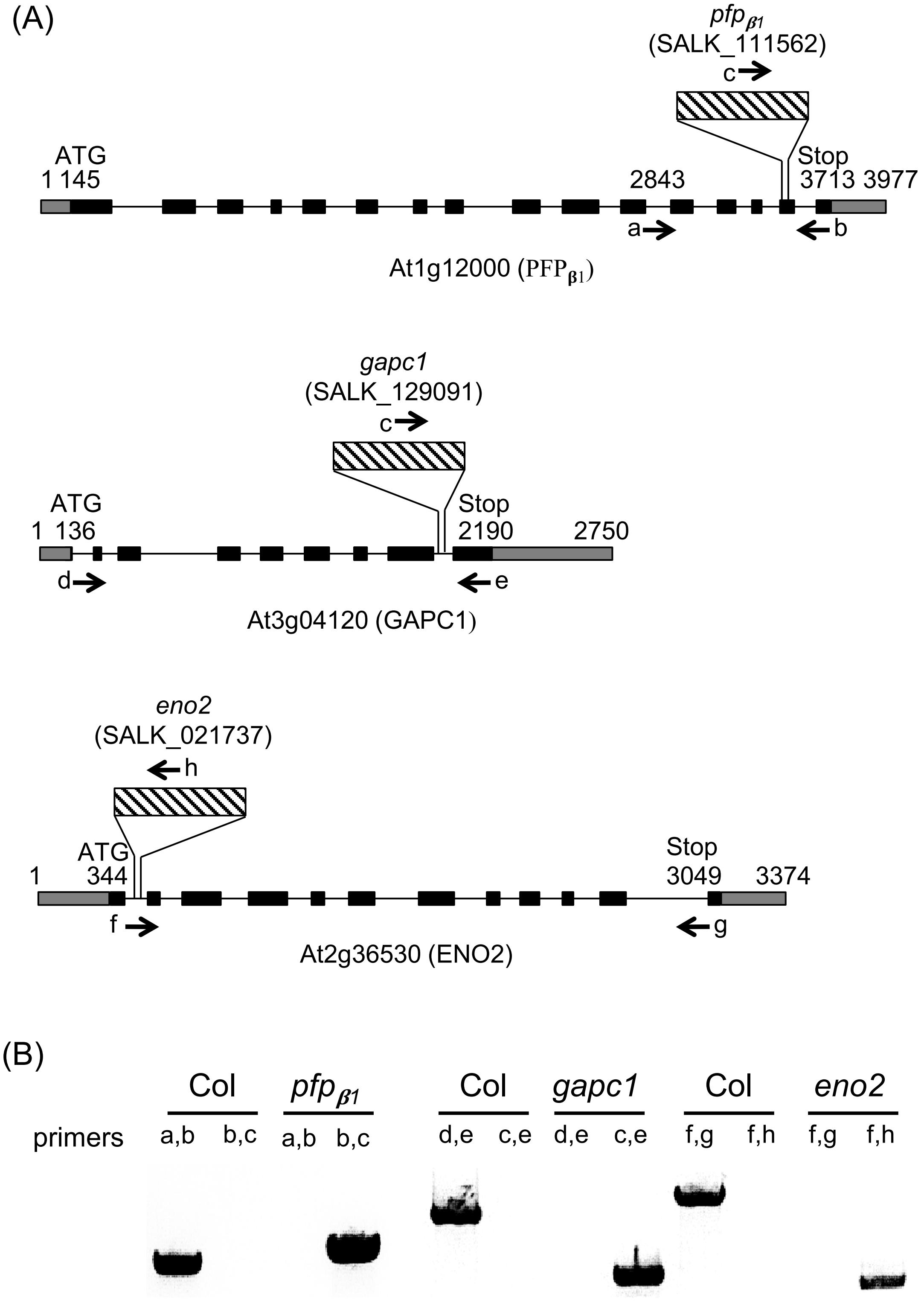

Figure S1
Genotyping of the $pfp_{\beta 1}$, *gapc1, eno2* T-DNA insertion lines. (A) Schematic diagram of T-DNA insertion sites at the $PFP_{\beta 1}$, *GAPC1*, *ENO2* loci. Grey box, black box, and black line indicate untranslated regions, coding regions, and introns, respectively. Arrows indicate the locations of the primers used for genotype analysis. (B) Genotype analysis of the $pfp_{\beta 1}$, *gapc1*, *eno2* lines. The location of the primers is shown in (A).

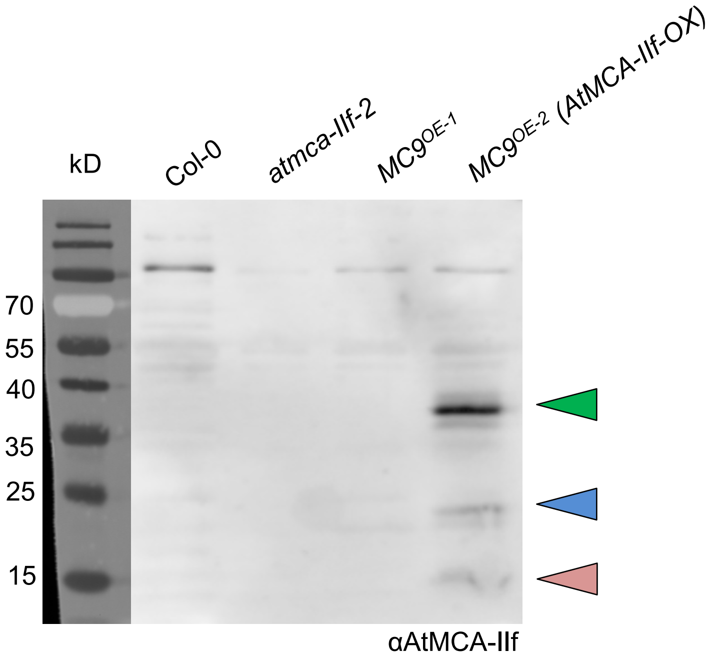

Figure S2
Immunoblot analysis of AtMCA-IIf in crude protein extracts from fifth leaves of four-week-old plants incubated in darkness for two days. Proteins were separated by SDS-PAGE and detected using an anti-AtMCA-IIf antibody. The full-length AtMCA-IIf protein is indicated by a green arrow, and lower-molecular-weight bands corresponding to autoprocessed AtMCA-IIf fragments are indicated by blue and pink arrows.

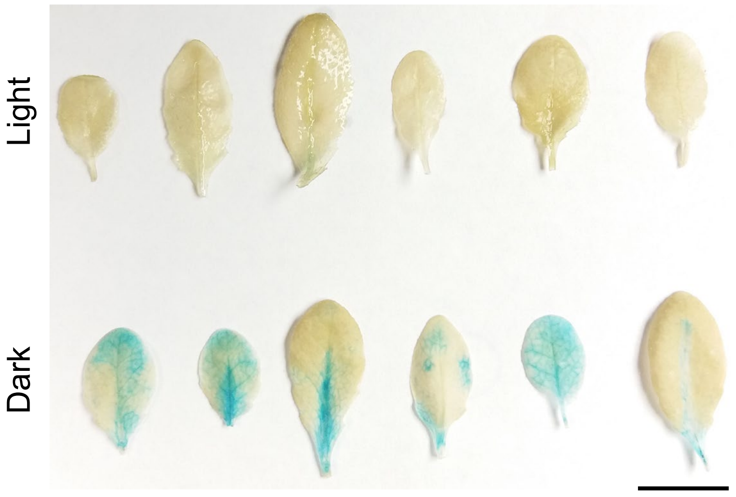

Figure S3
The activity of the *AtMCA-IIf* promoter in leaves treated in either light or darkness. A detached rosette leaves (fourth to sixth) from four-week-old plants was kept either in normal light conditions or in darkness for three days according to Nagahage et al. (2020). The blue color indicates the activity of the 1410-bp *AtMCA-IIf* promoter fused to β-glucuronidase (GUS) reporter gene in a transgenic pro*AtMCA-IIf*::GUS T2.10.5 line. Bar = 1 cm.

(A)

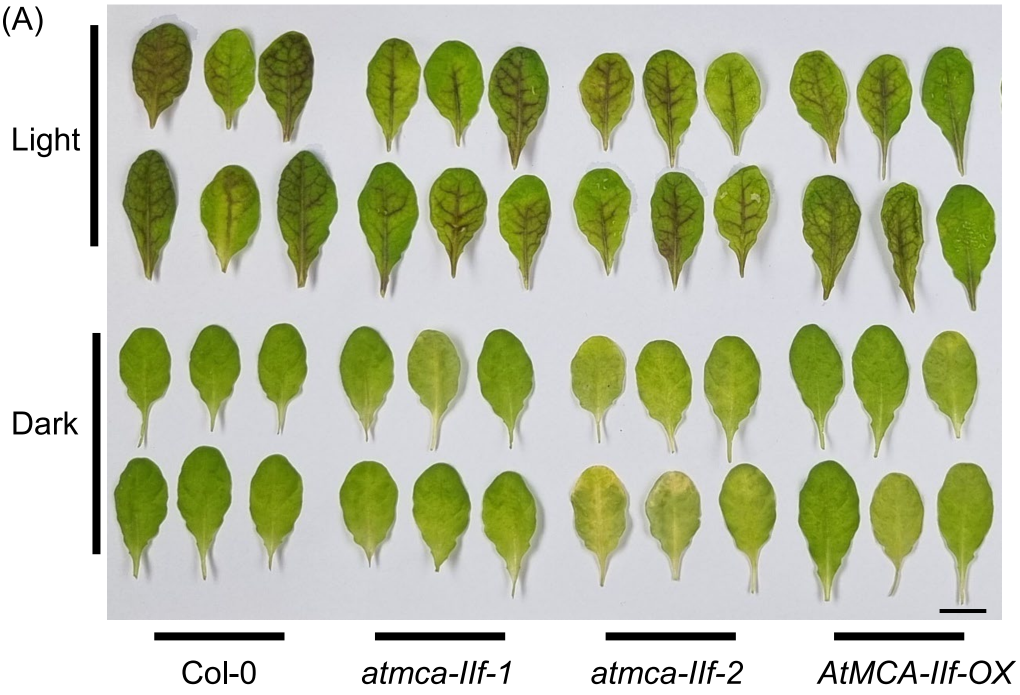

(B)

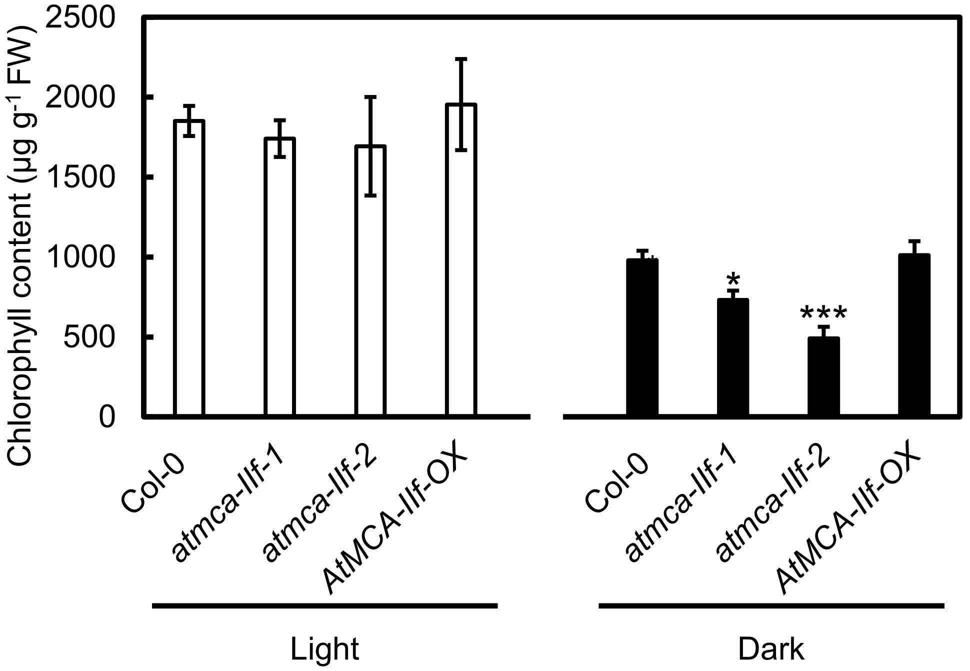

Figure S4

The function of AtMCA-IIf in leaves incubated in darkness and in continuous light. (A) The progress of senescence in detached leaves (the fifth leaf) of four-week-old *AtMCA-IIf-OX* and *atmca-IIf* plants incubated under continuous light or dark conditions for four days. Bar = 1 cm. (B) Chlorophyll content of the 5th leaf of four-week-old plants incubated under continuous light or dark conditions for four days. Values and error bars indicate means ± SE (n = 5 biologically independent replicates). Asterisks indicate means that are significantly different from the Col-0 wild type (Welch's *t*-test (two-tailed) *** $P < 0.001$, *$P < 0.05$).

(A)

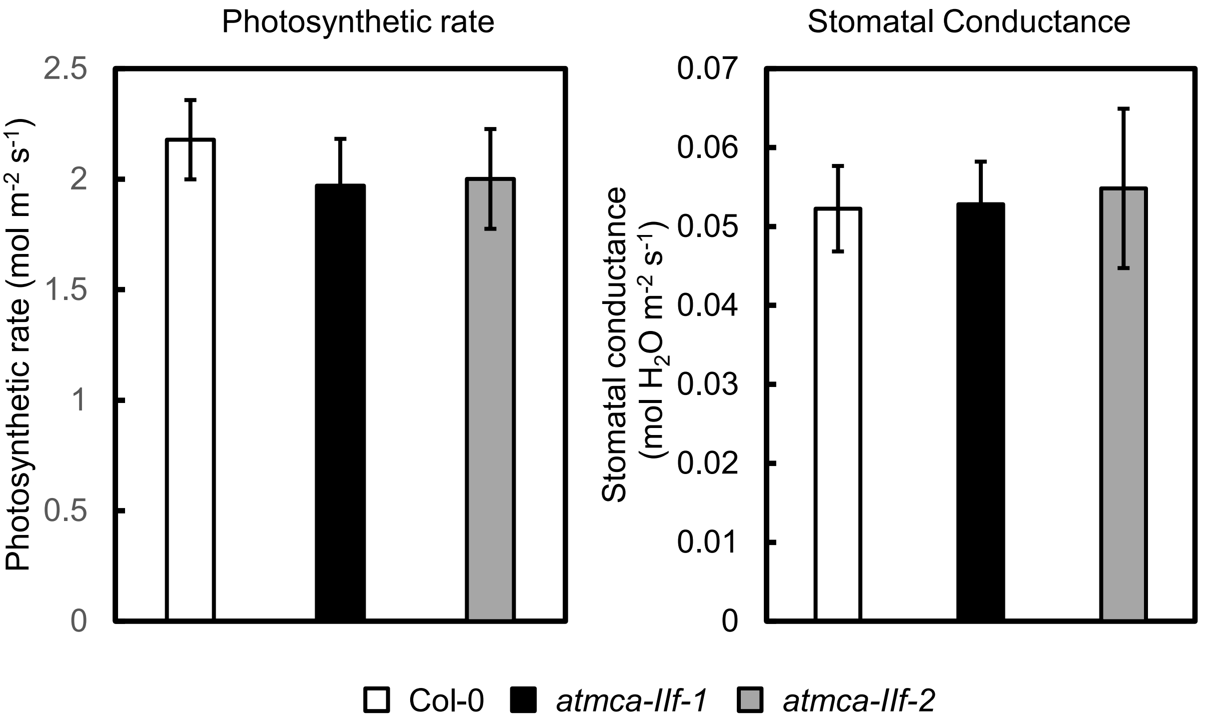

(B)

Figure S5

Photosynthetic rate and stomatal conductance of plants under light conditions. Data are presented as means of six independent biological replicates ± SE. Measurements were performed using a portable CO_2 infrared gas analyzer (LI-6400XT, LI-COR Environmental, USA) under chamber conditions of 1,000 μmol photons m^{-2} s^{-1} irradiance, 20°C, 400 μmol mol^{-1} CO_2, and a flow rate of 250 cm^3 min^{-1}. Measurements were taken from the fifth leaf of 4-week-old plants grown under long-day conditions (16/8 h light/dark) at 22 °C, 4 h after the start of the light cycle.

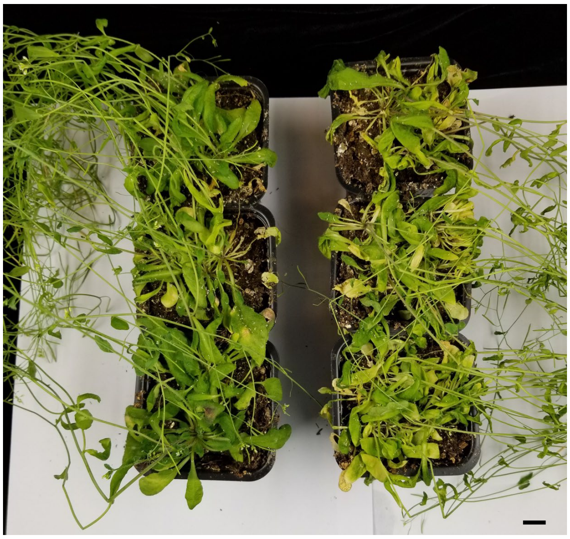

Col-0 *atmca-IIf-2*

Figure S6
Effect of *AtMCA-IIf* on age-dependent leaf senescence. Wild-type (Col) and *atmca-IIf-2* plants grown under long-day conditions for 45 days. The plants were exposed to mild drought stress by reducing the frequency of watering to every five days. Bar = 1 cm.

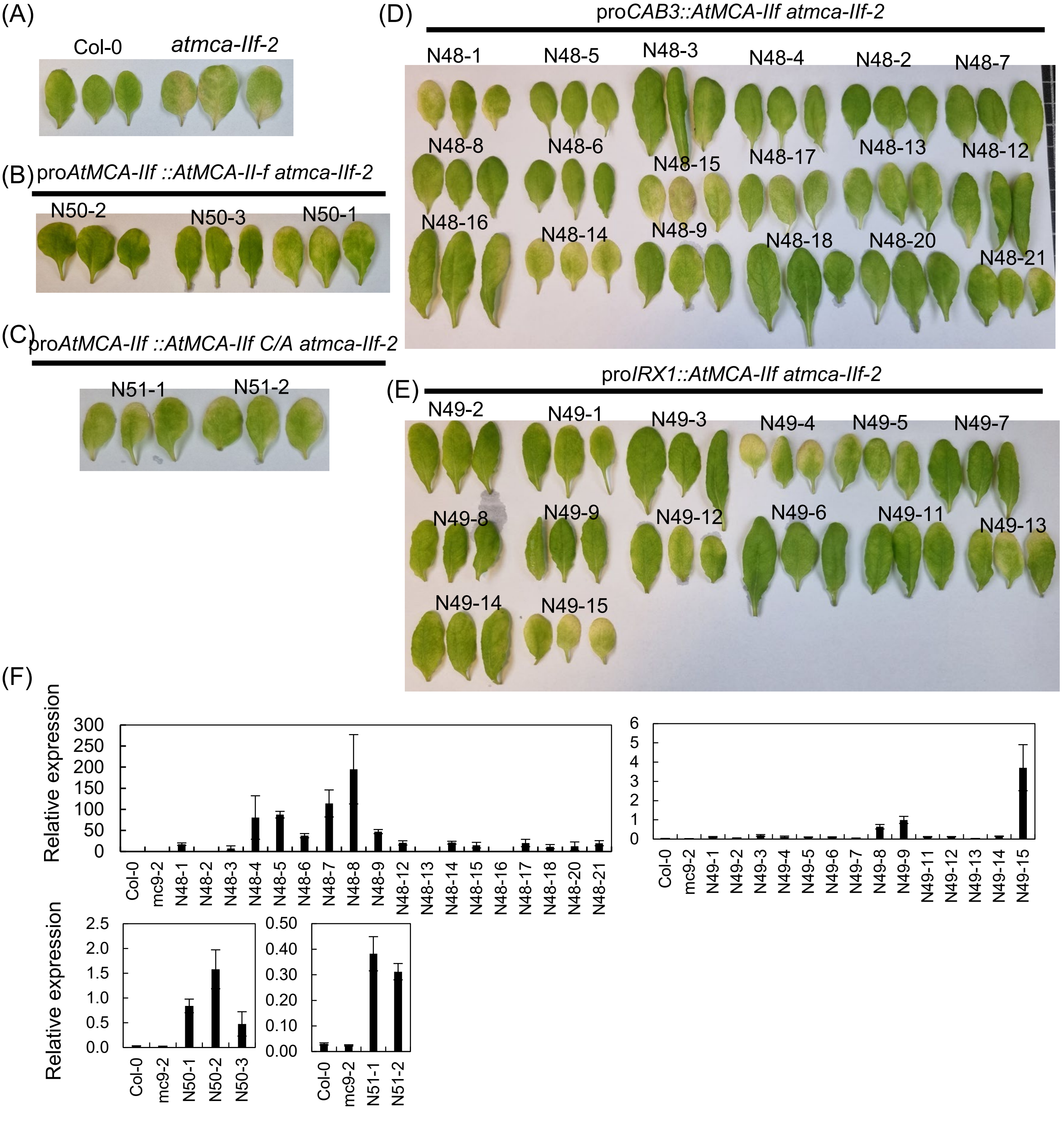

Figure S7

Leaf senescence in genetic complementation lines of the *atmca-IIf-2* mutant. The progress of senescence in detached leaves (the fifth leaf) of four-week-old plants incubated for four days under dark conditions in Col-0 wild type and *atmca-IIf-2* mutant (A), three independent lines carrying the pro*AtMCA-IIf::AtMCA-IIf* construct (B), two independent lines carrying the construct with the enzymatically inactive pro*AtMCA-IIf::AtMCA-IIf*-C/A (C)*,* 18 independent lines carrying the pro*CAB3::AtMCA-IIf* construct (D), and 14 independent lines carrying the pro*IRX1::AtMCA-IIf* construct (E) *in the atmca-IIf-2* mutant background. Bar = 1 cm. (F) The expression levels of *AtMCA-IIf* in the complementation lines. Quantitative RT-PCR analysis was performed using the dark-incubated fifth leaf of four-week-old plants.

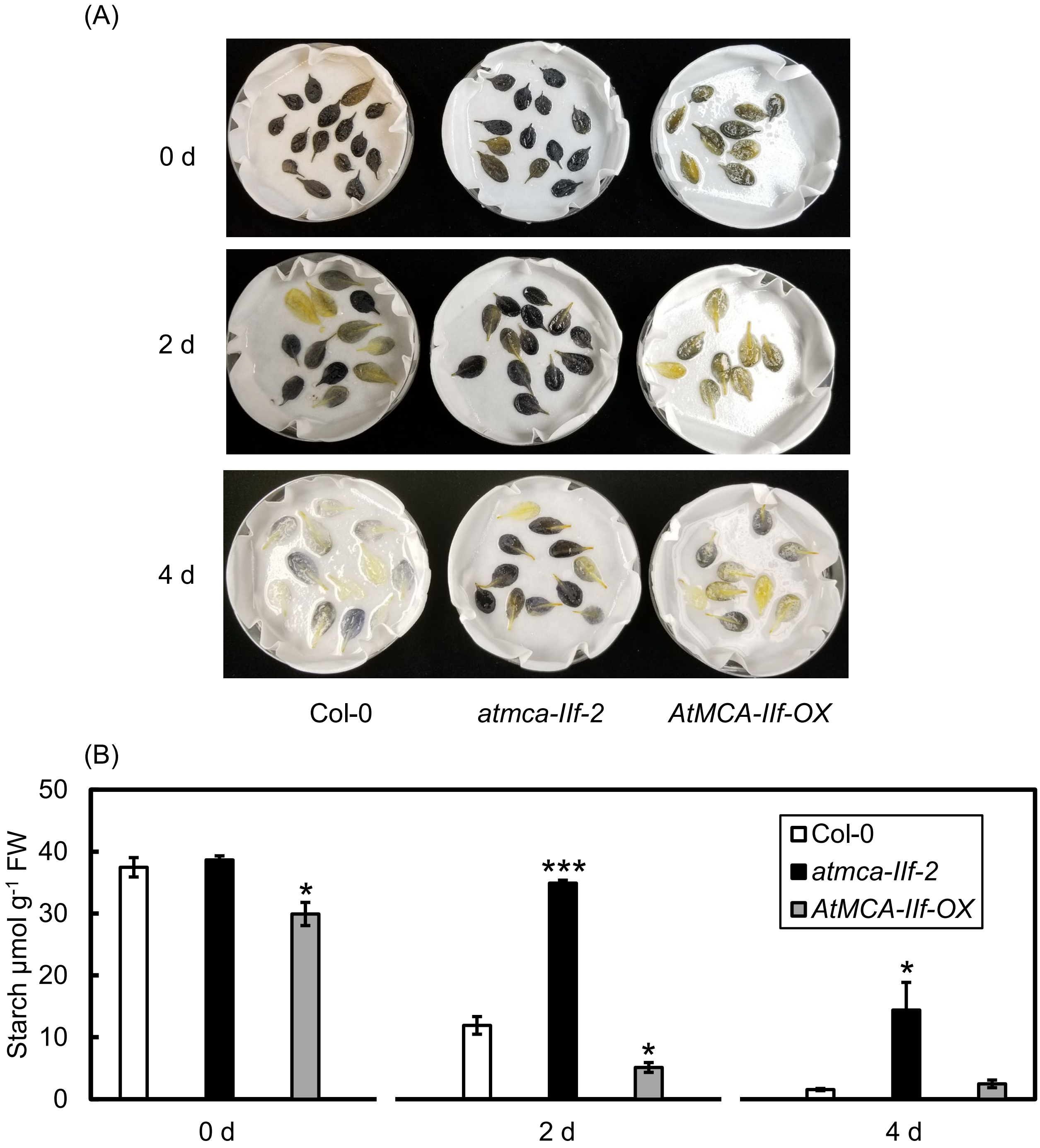

Figure S8

The effect of AtMCA-IIf on starch content of leaves before and after the dark treatment. (A) Iodine staining of starch in the leaves. (B) The starch content. Detached leaves of four-week-old *atmca-IIf* mutant, *AtMCA-IIf-OX* and Col-0 wild-type plants were analysed in light (0 day) and after two and four days of dark incubation. Values and error bars indicate means ± SE (n = 3 biologically independent replicates). Asterisks indicate means that are significantly different from the Col-0 plants (Welch's *t*-test (two-tailed) *** $P < 0.001$, * $P < 0.05$).

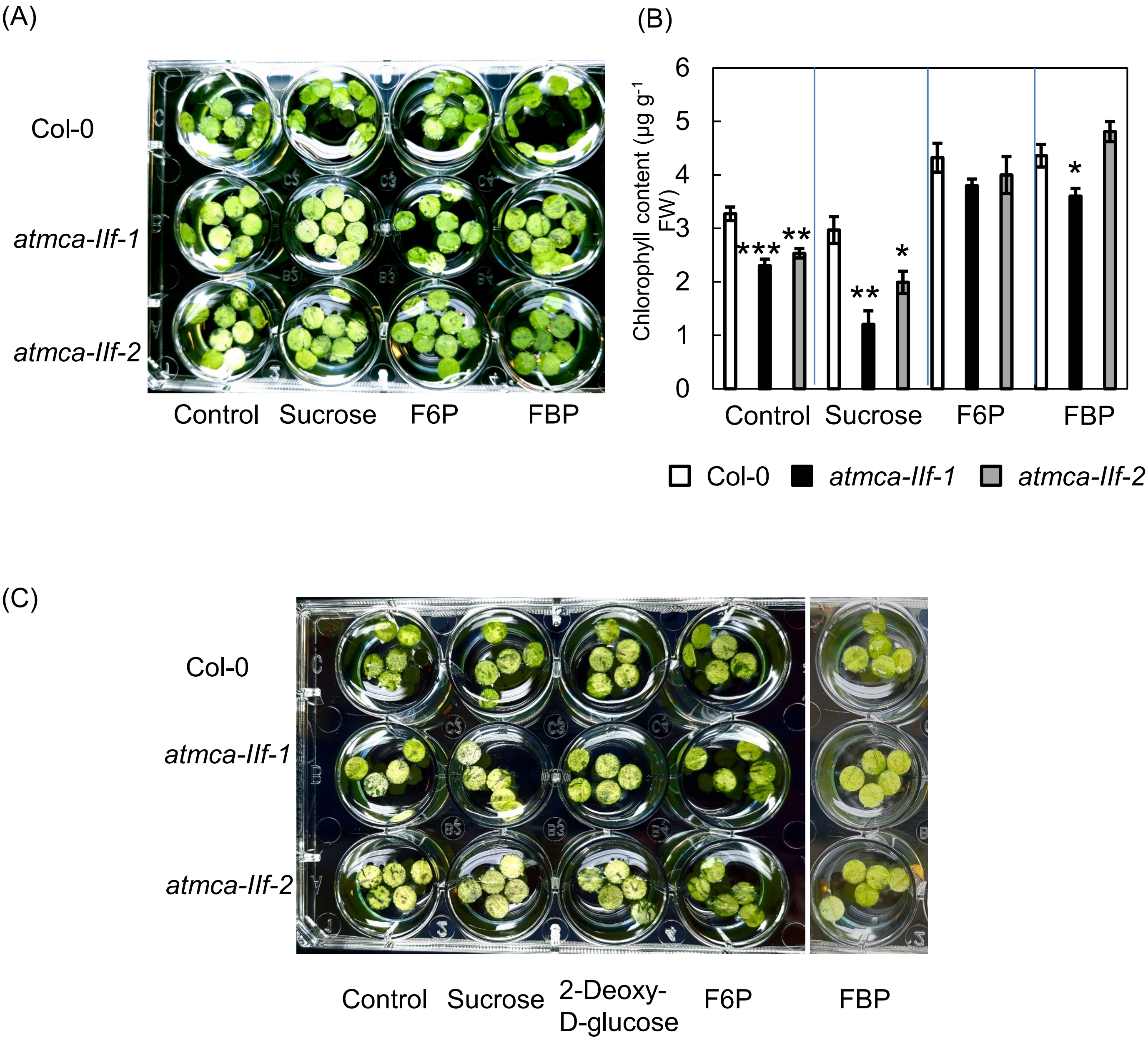

Figure S9

The effect of fructose phosphates on the accelerated senescence phenotype of *atmca-IIf* in darkness. The accelerated senescence phenotype of *atmca-IIf* was rescued by incubation of leaf discs from the fifth leaf of 4- to 5-week-old plants with fructose 6-phosphate (F6P) and fructose 1,6-bisphosphate (FBP) but accelerated by incubation with sucrose. The leaf discs were floated in phosphate buffer, pH 7, without any additional sugars (control) or with the addition of 50 mM sucrose, 50 mM F6P, or 50 mM FBP. Samples were kept in the dark at 25°C for three days, followed by visual inspection (A) and measurement of the total chlorophyll content (B). Asterisks indicate means that are significantly different from the Col-0 wild type (Welch's *t*-test (two-tailed) *** $P < 0.001$, ** $P < 0.01$, * $P < 0.05$). Values and error bars indicate means ± SE (n = 5 biologically independent replicates).

(C) The effect of physiologically active and inactive sugars on the accelerated senescence phenotype of *atmca-IIf* mutants in darkness. 2-Deoxy-D-glucose, which cannot be utilized in glycolysis, and sucrose did not revert but rather seemed to exacerbate the senescence phenotype of *atmca-IIf*, while F6P treatment rescued the *atmca-IIf* phenotype.
